# Building Community Health Literacy With a Social Network Perspective

**DOI:** 10.3928/24748307-20250224-01

**Published:** 2025-07

**Authors:** Tetine Sentell, Meliza Roman, Kara Saiki, Mary Ann Abrams, Alexandria Jones, Krizia Melendez, Jessica Chevrolet, Allison Riggle, Emily Szollosy, Opal Vanessa Buchthal

## Abstract

Advancing Health Literacy Franklin County was initiated in June 2021 with a goal of building a sustainable health literate community in Franklin County, Ohio, USA. The project was a collaboration among public health departments, health care organizations, an academic institution, and community organizations and included capacity-building over a 2-year period. Project evaluation included social network analysis mapping of organizational relationships over two project time periods to document change in this area. This brief report describes the social network analysis mapping as a project outcome, a valuable approach to advancing organizational health literacy and health equity, and a practical tool for other health literacy projects as sustainable relationships and networks are important in health literacy research and practice. [***HLRP: Health Literacy Research and Practice*. 2025;9(3):e78–e82.**]

The coronavirus disease 2019 (COVID-19) pandemic underscored the importance of health literacy and reliable information networks to provide guidance, combat misinformation, increase trust, and ensure health equity (Sentell et al., 2020). Advancing Health Literacy Franklin County (AHLFC) was initiated in June 2021 to build a sustainable health literate community in Franklin County, Ohio, USA (Upreti et al., 2023). The project, a collaboration among public health departments, health care organizations, an academic institution, and community organizations, included capacity-building over a 2-year period. The project was concordant with Healthy People 2030 health equity goals and guided by the definition of organizational health literacy as a collective responsibility: “the degree to which organizations equitably enable individuals to find, understand, and use information and services to inform health-related decisions and actions for themselves and others” (U.S. Department of Health and Human Services, 2020a).

The project had two goals: (1) Develop community and health literacy-informed COVID-19 messages; (2) Create and support a sustainable health literacy infrastructure for community organizations to reduce health disparities and promote health equity. Goals worked synergistically to build organizational capacity and networks. The expectation was a stronger community-level information ecosystem grounded in organizational health literacy, culturally relevant communications, and sustainable relationships (Upreti et al., 2023).

Evaluation included a social network analysis (SNA) of organizational relationships over time to understand relationships, resources, and impact of partnerships emerging throughout this project. SNA is a method for characterizing relationships within and across networks and can provide insights on collaboration changes. SNA is a useful tool for project evaluation, providing reliable information on how information and resources are shared within organizational networks, due to its ability to map the impacts of partnership-building initiatives over time. SNA can be used to strengthen collaborations by identifying both key players and potential partners. Additionally, SNA can document the impact of connecting organizations in facilitating communication, collaboration, and partnership development between/across diverse organizations (Harris et al., 2008).

This brief report describes the SNA mapping of organizations engaged in this regional effort.

## Methods

Organizational relationships were measured with an online REDCap (Research Electronic Data Capture) survey using standard SNA measures at two time points sent to individuals from organizations identified by project leads as engaged in AHLFC efforts at that time (Harris et al., 2009). Questions included the identification of collaborating organizations engaging in the health literacy initiative and ties in these relationships as measured by the following questions: (1) Please choose the response that best describes the current relationship between your organization and each of these organizations about health literacy (scale: 1 = *we do not interact* to 7 = *we plan together and share staff and funding*); (2) In the past year, how often did your agency have contact (meetings, phone calls, emails) with each of the following agencies about health literacy? (scale: 1 = *never* to 6 = *daily*); (3) How much would you say that your organization's overall goals about health literacy are shared with each of the following organizations? (scale: 1 = *we do not have any goal in common* to 4 = *we share all of the same goals*).

The survey was first sent from September 2022 to December 2022 (T1). The second time (T2) was from March 2023 to May 2023. Each time point survey received at least five email pushes from project leads. Responses were averaged if multiple respondents from an organization participated.

Data analysis and visualizations were prepared using R software version 4.2.0 (R Foundation for Statistical Computing, Vienna, Austria) using the packages tidyverse (Wickham et al., 2019), ggraph (Pedersen, 2024), and igraph (Csárdi & Nepusz, 2005). Network graphs were constructed with color-coded nodes representing organizations and organization type and edges representing collaborations between organizations based on survey responses. Betweenness centrality (the extent to which one organization operates as a focal point connecting the other organizations within the network) and graph “diameter,” the longest shortest walk between two nodes, (McNulty, 2022) were also measured. This project was exempt from human subject review as evaluation of a public health initiative.

## Results

AHLFC had 83% growth in engaged organizations from T1 to T2 (from 43 to 79 organizations) who received the survey push. Organization response rates were 55.8% (*N* = 24/43) for T1 and 21.5% (*N* = 17/79) for T2. At T2, 5 of 17 were new organizations, and 12 repeated from T1.

**Figure 1.**
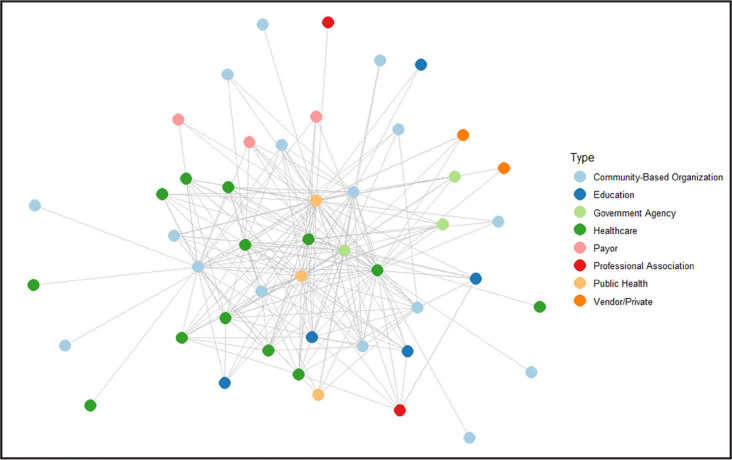
Organizations by type, time 1 and time 2. Overall network – time 1.

**Figure 2.**
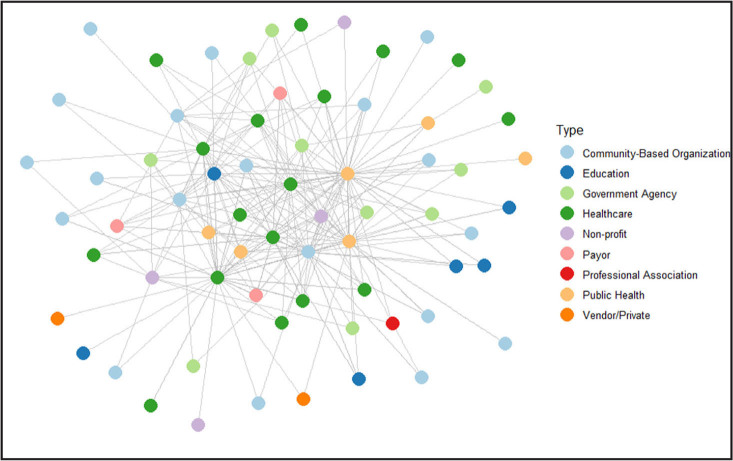
Overall network – time 2. There was a statistically significant (*p* < .01) difference in the number of nodes between the two graphs based on the Wilcoxon rank-sum test.

**Figure 1** shows the network graphs at T1 and T2 by organization type. There was a statistically significant (*p* < .01) difference in the number of nodes between the two graphs based on the Wilcoxon rank-sum test. T1 reflects 29 responses received from 24 unique organizations (**Figure 2**). T2 reflects 35 responses received from 17 unique organizations. The project lead organizations (Franklin County Public Health, Columbus Public Health, and Nationwide Childrens' Hospital) are centered in the middle of these networks as public health, public health, and health care organizations, respectively.

Various organization types were represented such as community-based, health care, education, government agency, payor, public health, professional, vendor/private, and non-profit. At each time point, over half of the participating organizations were community-based organizations and health care organizations. An increase over time was seen in engagement by community-based organizations (+3), health care organizations (+4), government agency organizations (+7), non-profit (+4), and public health associations (+3) among others.

Analyses revealed strengthening of relationships. Among overall organization connections, there was a 6% increase from T1 to T2 of collaborators who contact each other about health literacy quarterly. An increase from 41% at T1 to 50% at T2 of overall connections said they “shared some of the same goals” about health literacy. T2 had an increase of collaborators who contacted each other quarterly, from 23% to 26%. At T2, about 50% of overall connections responded that they share some of the same health literacy goals (increased from 41% at T1). At each time point, two project lead organizations had high betweenness centrality measures in network graphs, an indicator of their intermediary role in connecting organizations. Graph diameter was 4 at both time points.

## Discussion

Advancing community-level health literacy can be a valuable component of eliminating health disparities, achieving health equity, and attaining health literacy to improve the health and well-being of all (U.S. Department of Health and Human Services, 2020b). Public health practice, health care, and community organizations have shared goals to achieve health literate communities, but practice implementation, evaluation, and measurement on this topic at community levels of impact are still emerging (Baur et al., 2018). Social network analysis can be useful for this.

Results show growth in the number of engaged organizations, increase in diversity of organizations across sectors, and stronger ties in relationships from this initiative. This is particularly important as health is largely impacted by social determinants and population-focused efforts (Whitman et al., 2022). Furthermore, “health literacy is one modifiable factor mediating the link between socioeconomic status and health disparities” (Coleman et al., 2023).

Our findings demonstrate success in fostering connections between clinical, public health, and community-based and similar organizations to address organizational health literacy. Supported with organizational capacity-building to sustain internal expertise, enduring materials and resources, and evaluation tools, these networks have the potential for lasting community health impact (Stormacq et al., 2019).

We found growth in number and density of organization relationships, but did not see a noticeable change in the distance organizations were communicating, suggesting that the network was already fairly close-knit, and able to maintain closeness over time. When a network diameter is small, nodes are tightly interconnected, and information flow is likely to be fast. This can be valuable in dynamic information situations like public health emergencies.

Network studies provide unique insight into the growth of relationships over time (Buchthal & Maddock, 2015; Jeffries et al., 2019). While documenting the impact of collaborative work is a useful evaluative activity in itself, network data can be even more useful when shared and jointly analyzed with partners. Network graphs are particularly effective at visualizing the invisible, helping community members see and discuss the structure of existing connections. Discussions can help partners lean into the network's emerging strengths, find organizations missed in their efforts to share information or build collaboration, and strategize ways to strengthen bonds with partners who are poorly engaged. While sophisticated SNA tools exist, basic analyses methods, including visualizations, are straightforward in R programming and other open-source SNA and visualization software and should be accessible as practical tools for many public health projects (Derr, 2024). These tools and methods are valuable yet underused (Leppin et al., 2018).

## Conclusion

SNA maps develop an asset-based community framework while also identifying groups not yet at the table, promoting equity by addressing gaps and ensuring all voices are heard, topics of high relevance to health literacy initiatives. Comparing these data to demographic and geographic data from sources such as Policy Map could identify needs in locations not yet being reached. SNA mapping also helps avoid duplication of efforts and maximizes use of local resources, leading to more sustainable outcomes. Information can also be used to establish formal community collaboratives to leverage resources and develop new initiatives. Additionally, involving partners in the mapping process promotes a sense of ownership and accountability. Network maps can be valuable to demonstrate community-wide commitment to specific issues for policy action and can enhance grant applications. Finally, social network mapping is asset-rather than deficit-based, re-focusing attention from problems to opportunities, and empowering communities with a collectivist perspective.

Practical lessons include the challenges to full SNA response rates. While our analysis shows impact, lack of participation from all organizations and possible nonresponse bias cause the full network to not be visible and impact may be greater than shown. Networks may reflect only the interactions between more active organizations, missing important collaborations. Also, there were many evaluation activities, so respondents may have experienced survey fatigue, which may explain the lower response rates for T2 SNA; this would not necessarily be true for other projects. Another limitation is that the data for network relationships are from a limited assessment of one report from each organization and accuracy of each reporter may also vary as one individual may not be aware of all organizational collaborations, particularly in large organizations.

Considering health literacy in a social context is important to building sustainable community-level organizational health literacy, including networks and engaging community leaders to share trusted, relevant health information in professional and personal social networks (Upreti et al., 2023). Insights from this real-world implementation can inform future practice, policy, and research, including planning similar projects to achieve robust SNA response rates.

## References

[x24748307-20250224-01-bibr1] Baur , C. , Martinez , L. M. , Tchangalova , N. , & Rubin , D . ( 2018 ). A review and report of community-based health literacy interventions. In National Academies of Sciences, Engineering, and Medicine; Health and Medicine Division; Board on Population Health and Public Health Practice; Roundtable on Health Literacy . *Community-based health literacy interventions: Proceedings of a workshop* . National Academies Press . https://www.ncbi.nlm.nih.gov/sites/books/NBK500372/ 29543418

[x24748307-20250224-01-bibr2] Buchthal , O. V. , & Maddock , J. E. ( 2015 ). Mapping the possibilities: Using network analysis to identify opportunities for building nutrition partnerships within diverse low-income communities . *Journal of Nutrition Education and Behavior* , *47* ( 4 ), 300 – 307.e1 . 10.1016/j.jneb.2015.03.002 PMID: 25864890

[x24748307-20250224-01-bibr3] Coleman , C. , Birk , S. , & DeVoe , J. ( 2023 ). Health Literacy and systemic racism-using clear communication to reduce health care inequities . *JAMA Internal Medicine* , *183* ( 8 ), 753 – 754 . 10.1001/jamainternmed.2023.2558 PMID: 37358860

[x24748307-20250224-01-bibr4] Csárdi , G. , & Nepusz , T. ( 2005 ). The igraph software package for complex network research . *InterJournal Complex Systems* , *1695* ( 5 ), 1 – 9 . https://www.researchgate.net/profile/Gabor-Csardi/publication/221995787_The_Igraph_Software_Package_for_Complex_Network_Research/links/0c96051d301a30f265000000/The-Igraph-Software-Package-for-Complex-Network-Research.pdf

[x24748307-20250224-01-bibr5] Derr , A . ( 2024 , February 14 ). *Social network analysis tools: 11 options for relationship mapping* . Visible Network Labs . https://visiblenetworklabs.com/2024/02/14/social-network-analysis-tools-for-mapping-relationships/

[x24748307-20250224-01-bibr6] Harris , J. K. , Luke , D. A. , Burke , R. C. , & Mueller , N. B. ( 2008 ). Seeing the forest and the trees: Using network analysis to develop an organizational blueprint of state tobacco control systems . *Social Science & Medicine* , *67* ( 11 ), 1669 – 1678 . 10.1016/j.socscimed.2008.07.013 PMID: 18722038

[x24748307-20250224-01-bibr7] Harris , P. A. , Taylor , R. , Thielke , R. , Payne , J. , Gonzalez , N. , & Conde , J. G. ( 2009 ). Research electronic data capture (REDCap)—A metadata-driven methodology and workflow process for providing translational research informatics support . *Journal of Biomedical Informatics* , *42* ( 2 ), 377 – 381 . 10.1016/j.jbi.2008.08.010 PMID: 18929686 PMC2700030

[x24748307-20250224-01-bibr8] Jeffries , N. , Zaslavsky , A. M. , Diez Roux , A. V. , Creswell , J. W. , Palmer , R. C. , Gregorich , S. E. , Reschovsky , J. D. , Graubard , B. I. , Choi , K. , Pfeiffer , R. M. , Zhang , X. , & Breen , N. ( 2019 ). Methodological approaches to understanding causes of health disparities . *American Journal of Public Health* , *109* ( S1 ), S28 – S33 . 10.2105/AJPH.2018.304843 PMID: 30699015 PMC6356121

[x24748307-20250224-01-bibr9] Leppin , A. L. , Okamoto , J. M. , Organick , P. W. , Thota , A. D. , Barrera-Flores , F. J. , Wieland , M. L. , McCoy , R. G. , Bonacci , R. P. , & Montori , V. M. ( 2018 ). Applying social network analysis to evaluate implementation of a multisector population health collaborative that uses a bridging hub organization . *Frontiers in Public Health* , *6* , 315 . 10.3389/fpubh.2018.00315 PMID: 30450355 PMC6224340

[x24748307-20250224-01-bibr10] McNulty , K. ( 2022 ). Paths and distance . In *Handbook of graphs and networks in people analytics: With examples in R and Python* (pp. 119 – 145 ). Chapman and Hall/CRC . Retrieved April 8, 2024, from 10.1201/9781003266815-5

[x24748307-20250224-01-bibr11] Pedersen , T. L. ( 2024 , March 7 ). *ggraph: An implementation of grammar of graphics for graphs and networks* . The Comprehensive R Archive Network . https://cran.r-project.org/web/packages/ggraph/index.html

[x24748307-20250224-01-bibr12] Sentell , T. , Vamos , S. , & Okan , O. ( 2020 ). Interdisciplinary perspectives on health literacy research around the world: More important than ever in a time of COVID-19 . *International Journal of Environmental Research and Public Health* , *17* ( 9 ), 3010 . 10.3390/ijerph17093010 PMID: 32357457 PMC7246523

[x24748307-20250224-01-bibr13] Stormacq , C. , Van den Broucke , S. , & Wosinski , J. ( 2019 ). Does health literacy mediate the relationship between socioeconomic status and health disparities? Integrative review . *Health Promotion International* , *34* ( 5 ), e1 – e17 . 10.1093/heapro/day062 PMID: 30107564

[x24748307-20250224-01-bibr14] Upreti , R. , Saiki , K. , Abrams , M. A. , Jones , A. , Melendez , K. , Chevrolet , J. , Pennington , H. , Leadingham , A. , Martin , D. , & Sentell , T. ( 2023 ). Building community health literacy to achieve health equity: Insights from Ethiopian Tewahedo social services community leader in a county-level health literacy initiative . *Health Equity* , *7* ( 1 ), 592 – 597 . 10.1089/heq.2023.0069 PMID: 37731788 PMC10507930

[x24748307-20250224-01-bibr15] U.S. Department of Health and Human Services . ( 2020a ). *Health literacy in healthy people 2030* . https://health.gov/healthypeople/priority-areas/health-literacy-healthy-people-2030

[x24748307-20250224-01-bibr16] U.S. Department of Health and Human Services . ( 2020b ). *Healthy people 2030 framework* . https://health.gov/healthypeople/about/healthy-people-2030-framework

[x24748307-20250224-01-bibr17] Whitman , A. , De Lew , N. , Chappel , A. , Aysola , V. , Zuckerman , R. , & Sommers , B. D. ( 2022 ). *Addressing social determinants of health: Examples of successful evidence-based strategies and current federal efforts* . Assistant Secretary for Planning and Evaluation . https://aspe.hhs.gov/sites/default/files/documents/e2b650cd64cf84aae8ff-0fae7474af82/SDOH-Evidence-Review.pdf

[x24748307-20250224-01-bibr18] Wickham , H. , Averick , M. , Bryan , J. , Chang , W. , McGowan , L. D. A. , François , R. , Grolemund , G. , Hayes , A. , Henry , L. , Hester , J. , Kuhn , M. , Pedersen , T. L. , Miller , E. , Bache , S. M. , Müller , K. , Ooms , J. , Robinson , D. , Seidel , D. P. , Spinu , V. , Yutani , H. ( 2019 ). Welcome to the Tidyverse . *Journal of Open Source Software* , *4* ( 43 ), 1686 . 10.21105/joss.01686 .

